# Revisiting the Immune Frontier in Soft Tissue Sarcomas

**DOI:** 10.1007/s11912-026-01761-y

**Published:** 2026-03-17

**Authors:** Nadeem Bilani, Nourhane Al Akoum, Rusul Al-Marayaty, Sarah Orlando, Borislav Alexiev, Pedro Hermida de Viveiros, Seth M. Pollack

**Affiliations:** 1 Northwestern University Feinberg School of Medicine, Chicago, IL 60611 USA; 2 MedStar Washington Hospital Center, Washington, DC 20010 USA; 3 Medical Oncology, Fondazione Policlinico Universitario Campus Bio- Medico, Roma, 00128 Italy

**Keywords:** Soft tissue sarcoma (STS), Immuno-oncology, Immunotherapeutic strategies, Phase I–III clinical trials, Histology-specific responses, Forward-looking perspective, Trial design and therapeutic development, Outcomes

## Abstract

**Purpose of Review:**

Soft tissue sarcomas (STS) comprise a heterogeneous group of mesenchymal malignancies with limited treatment options and poor outcomes in the advanced setting. Although immune checkpoint inhibitors have transformed the management of many solid tumors, their efficacy in STS has been modest and strongly histology dependent. This review aims to synthesize recent advances in immuno-oncology as applied to STS and to highlight emerging strategies that may overcome resistance and improve patient outcomes.

**Recent Findings:**

Thus far, clinically meaningful activity of immune checkpoint inhibitors has been identified select STS subtypes, including undifferentiated pleomorphic sarcoma, angiosarcoma, and alveolar soft part sarcoma. Combination approaches incorporating immune checkpoint inhibitors with chemotherapy, radiation, tyrosine kinase inhibitors, or novel immune modulators have shown enhanced antitumor activity in early-phase and randomized trials. In parallel, engineered T-cell therapies targeting cancer-testis antigens have emerged as a standard-of-care option in synovial sarcoma and are being expanded to other histologies. Finally, advances in tumor microenvironment characterization, including the role of tertiary lymphoid structures and myeloid modulation, are refining patient selection and informing rational trial design.

**Summary:**

Immunotherapy continues to reshape the therapeutic landscape of soft tissue sarcoma. While immune checkpoint blockade alone benefits only a subset of patients, rational combination strategies and cellular therapies offer promising avenues to broaden clinical efficacy. Continued integration of biomarker-driven approaches, translational correlative studies, and histology-specific trial designs will be essential to fully realize the potential of immunotherapy in STS.

## Introduction

Soft tissue sarcoma (STS) constitutes a heterogenous group of mesenchymal malignancies, with over 50 histological subtypes [1]. The cornerstone of localized STS management remains surgical intervention, yet many patients present with advanced, unresectable disease for which systemic therapy is required. This may consist of single-agent chemotherapy, or combination regimens that are typically anthracycline-based [2].

**Fig. 1:**
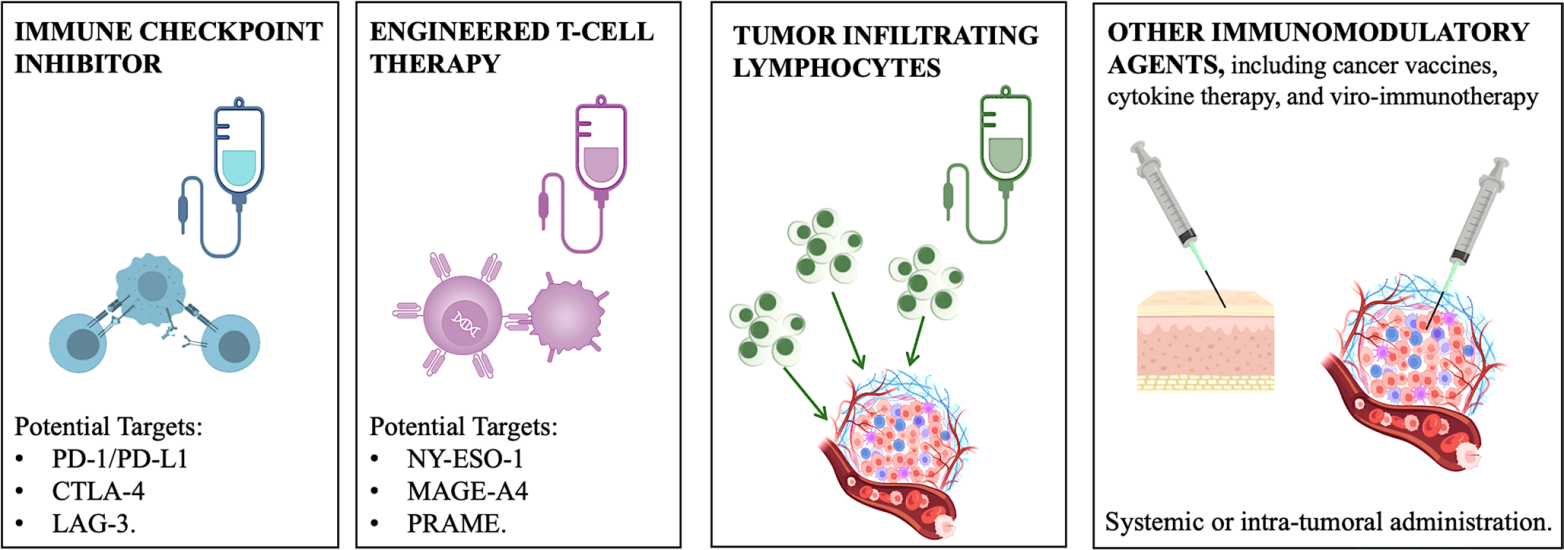
Forms of immunotherapy investigated in soft tissue sarcoma

Immunotherapy encompasses a broad range of treatment modalities, including immune checkpoint inhibitors (ICI), engineered T-cell therapies – such as chimeric antigen receptor T-cell therapy (CAR-T) – tumor-infiltrating lymphocyte (TIL) therapy, cancer vaccines, and tumor microenvironment (TME) modulating agents (Fig. 1). Sarcomas have historically been considered immunologically “cold” tumors, characterized by low immune cell infiltration and low tumor mutational burden. However, the application of immunotherapy in sarcomas is growing [3, 4].

At this time, the National Comprehensive Cancer Network (NCCN) recommends pembrolizumab as a subsequent-line treatment for certain subtypes of advanced or metastatic STS, including cutaneous angiosarcoma and undifferentiated STS such as myxofibrosarcoma (MFS) and undifferentiated pleomorphic sarcoma (UPS) [5]. Atezolizumab is recommended as one preferred front-line approach to the management of advanced alveolar soft part sarcoma (ASPS), and engineered T-cell therapy is now a standard part of treatment for appropriate patients with metastatic synovial sarcoma [6]. Numerous combination strategies, such as dual ICI therapy, ICI with chemotherapy, targeted agents, or other immunotherapies, are under active investigation to enhance disease control and prolong survival.

Given the rarity and heterogeneity of sarcoma subtypes, most clinical trials group these tumors together, or with other histologies, limiting our understanding of subtype-specific predictive biomarkers and therapeutic responsiveness. In this review, we aim to provide a comprehensive update on the current landscape of immunotherapeutic strategies across the diverse spectrum of STS histologies. Key clinical trials are summarized in Table 1..

**Table 1.** Key clinical trials evaluating immunotherapeutic approaches in soft tissue sarcoma

Therapeutic Approach	Author (Year)	Study Name	Clinical Trial Identifier
Immune checkpoint inhibitor monotherapy	Lakhani 2024	A first-in-human phase I study of the PD-1 inhibitor, retifanlimab (INCMGA00012), in patients with advanced solid tumors (POD1UM-101)	NCT03059823
	Nishikawa 2024	Efficacy and safety of nivolumab monotherapy in patients with unresectable clear cell sarcoma and alveolar soft part sarcoma (OSCAR Trial/NCCH1510)	NCCH1510
	Day 2023	A first-in-human phase 1 study of nofazinlimab, an anti-PD-1 antibody, in advanced solid tumors and in combination with regorafenib in metastatic colorectal cancer	NCT03475251
	Blay 2023	Pembrolizumab in patients with rare and ultra-rare sarcomas (AcS´e Pembrolizumab): analysis of a subgroup from a non-randomised, open-label, phase 2, basket trial	NCT03012620
	Chen 2023	Atezolizumab for advanced alveolar soft part sarcoma	NCT03141684
	Chawla 2022	Phase II randomized study of CMB305 and atezolizumab compared with atezolizumab alone in soft-tissue sarcomas expressing NY-ESO-1	NCT02609984
	Naing 2021	CX-072 (pacmilimab), a Probody^®^ PD-L1 inhibitor, in advanced or recurrent solid tumors (PROCLAIM-CX-072): an open-label dose-finding and first-in-human study	NCT03013491
	Shi 2020	Activity and safety of geptanolimab (GB226) for patients with unresectable, recurrent, or metastatic alveolar soft part sarcoma: a phase II, single-arm study	NCT03623581
	Yang 2020	Safety and clinical efficacy of toripalimab, a PD-1 mAb, in patients with advanced or recurrent malignancies in a phase I study	NCT02836834
	Tamura 2019	Efficacy and safety of nivolumab in Japanese patients with uterine cervical cancer, uterine corpus cancer, or soft tissue sarcoma: multicenter, open-label phase 2 trial	JapicCTI-163,212
	D’Angelo 2018	A non-comparative multi-center randomized phase II study of nivolumab +/− ipilimumab for patients with metastatic sarcoma (Alliance A091401)	NCT02500797
	Ben-Ami 2017	Immunotherapy with single agent nivolumab for advanced leiomyosarcoma of the uterus: results of a phase 2 study	NCT02428192
	Tawbi 2017	Pembrolizumab in advanced soft tissue and bone sarcomas: results of SARC028, a multicentre, single arm, phase 2 trial	NCT02301039
	Maki 2013	A pilot study of anti-CTLA4 antibody ipilimumab in patients with synovial sarcoma	NCT00140855
Immune checkpoint inhibitor with radiation therapy	Mowery 2024	Safety and efficacy of pembrolizumab, radiation therapy, and surgery versus radiation therapy and surgery for stage III soft tissue sarcoma of the extremity (SU2C-SARC032): an open-label, randomised clinical trial	NCT03092323
Dual immune checkpoint inhibitor therapy	ONGOING	ENVASARC: A pivotal trial of envafolimab and envafolimab in combination with ipilimumab in patients with advanced or metastatic undifferentiated pleomorphic sarcoma or myxofibrosarcoma who have progressed on prior chemotherapy.	NCT04480502
	Wilky 2025	Botensilimab (Fc-enhanced anti-cytotoxic lymphocyte-association protein-4 antibody) Plus Balstilimab (anti-PD-1 antibody) in Patients With Relapsed/Refractory Metastatic Sarcomas	NCT03860272
	Roland 2024	Nivolumab With and Without Ipilimumab and Radiation Therapy in Treating Patients With Recurrent or Resectable Undifferentiated Pleomorphic Sarcoma or Dedifferentiated Liposarcoma Before Surgery	NCT03307616
	Chae 2024	Phase II basket trial of dual anti-CTLA-4 and anti-PD-1 blockade in rare tumors (DART) SWOG S1609: the desmoid tumors	NCT02834013
	Somaiah 2022	Durvalumab plus tremelimumab in advanced or metastatic soft tissue and bone sarcomas: a singlecentre phase 2 trial	NCT02815995
	Wagner 2021	Multicenter phase II trial (SWOG S1609, cohort 51) of ipilimumab and nivolumab in metastatic or unresectable angiosarcoma: a substudy of dual anti-CTLA-4 and anti-PD-1 blockade in rare tumors (DART)	NCT02834013
	D’Angelo 2018	A non-comparative multi-center randomized phase II study of nivolumab +/− ipilimumab for patients with metastatic sarcoma (Alliance A091401)	NCT02500797
Immune checkpoint inhibitor with tyrosine kinase inhibitor	Grilley-Olson 2024	A multicenter phase II study of cabozantinib + nivolumab for patients (pts) with advanced angiosarcoma (AS) previously treated with a taxane (Alliance A091902)	NCT04339738
	Movva 2024	Histology-Specific Clinical Trial of Lenvatinib and Pembrolizumab in Patients with Sarcoma	NCT04784247
	Cho 2024	Durvalumab plus pazopanib combination in patients with advanced soft tissue sarcomas: a phase II trial	NCT03798106
	Van Tine 2023	Cabozantinib Combined With PD-1 and CTLA-4 Inhibition in Metastatic Soft Tissue Sarcoma	NCT04551430
	Liu 2022	Phase II study of TQB2450, a novel PD-L1 antibody, combination with anlotinib in patients with locally advanced or metastatic soft tissue sarcoma	NCT03897283
	Broto 2020	Nivolumab and sunitinib combination in advanced soft tissue sarcomas: a multicenter, singlearm, phase Ib/II trial	NCT03277924
	Wilky 2019	Axitinib plus pembrolizumab in patients with advanced sarcomas including alveolar soft-part sarcoma: a single-centre, single-arm, phase 2 trial	NCT02636725
Immune checkpoint inhibitor with cyclin dependent kinase inhibitor	ONGOING	A phase II study of palbociclib combined with retifanlimab in patients with advanced dedifferentiated liposarcoma.	NCT04438824
	ONGOING	Testing the Addition of Cemiplimab to Palbociclib for the Treatment of Advanced Dedifferentiated Liposarcoma (A092107)	NCT05694871
Immune checkpoint inhibitor with chemotherapy	Haddox 2024	Phase II study of eribulin plus pembrolizumab in metastatic soft-tissue sarcomas: clinical outcomes and biological correlates	NCT03899805
	Tian 2024	Combining nanoparticle albumin-bound paclitaxel with camrelizumab in advanced soft tissue sarcoma: activity, safety, and future perspectives	NCT05189483
	Gordon 2023	SAINT: A Phase I/expanded phase II study using safe amounts of Ipilimumab, Nivolumab and Trabectedin as first-line treatment of advanced soft tissue sarcoma	NCT03138161
	Tian 2022	Efficacy and safety of sintilimab plus doxorubicin in advanced soft tissue sarcoma: a single-arm, phase II trial	NCT04356872
	Toulmonde 2022	Trabectedin plus durvalumab in patients with advanced pretreated soft Tissue Sarcoma and Ovarian Carcinoma (TRAMUNE): an open-label, multicenter phase Ib study	NCT03475953
	Wagner 2022	A phase 1/2 trial combining avelumab and trabectedin for advanced liposarcoma and leiomyosarcoma	NCT03074318
	Italiano 2022	Pembrolizumab in soft-tissue sarcomas with tertiary lymphoid structures: a phase 2 PEMBROSARC trial cohort	NCT02406781
	Pollack 2020	Assessment of doxorubicin and pembrolizumab in patients with advanced anthracycline-naive sarcoma	NCT02888665
	Toulmonde 2018	Use of PD-1 targeting, macrophage infiltration, and IDO pathway activation in sarcomas	NCT02406781
Immunomodulatory agents with or without immune checkpoint inhibitor therapy	Toulmonde 2024	Reshaping the tumor microenvironment of cold soft-tissue sarcomas with oncolytic viral therapy: a phase 2 trial of intratumoral JX-594 combined with avelumab and low-dose cyclophosphamide	NCT02630368
	Seo 2023	Toll-Like Receptor 4 Agonist Injection With Concurrent Radiotherapy in Patients With Metastatic Soft Tissue Sarcoma	NCT02180698
	Chawla 2023	Activity of TNT: a phase 2 study using talimogene laherparepvec, nivolumab and trabectedin for previously treated patients with advanced sarcomas	NCT03886311
	Kelly 2023	A phase 2 study of epacadostat and pembrolizumab in patients with advanced sarcoma	NCT03085914
	Zhou 2023	A pilot study of multi-antigen stimulated cell therapy-I plus camrelizumab and apatinib in patients with advanced bone and soft-tissue sarcomas	NCT04074564
	Xie 2023	Exploratory study of an anti-PD-L1/TGF-β antibody, TQB2858, in patients with refractory or recurrent osteosarcoma and alveolar soft part sarcoma: a report from Chinese sarcoma study group (TQB2858-Ib-02)	CTR20220390
	Chawla 2022	Phase II randomized study of CMB305 and atezolizumab compared with atezolizumab alone in soft-tissue sarcomas expressing NY-ESO-1	NCT02609984
	Kelly 2020	Objective response rate among patients with locally advanced or metastatic sarcoma treated with talimogene laherparepvec in combination with pembrolizumab	NCT03069378
	Zhang 2019	Systemic Interferon-γ Increases MHC Class I Expression and T-cell Infiltration in Cold Tumors: Results of a Phase 0 Clinical Trial	NCT01957709
Cellular therapy	D’Angelo 2025	Letetresgene Autoleucel in Advanced/Metastatic Myxoid/Round Cell Liposarcoma	NCT02992743
	D’Angelo 2024	Afamitresgene autoleucel for advanced synovial sarcoma and myxoid round cell liposarcoma (SPEARHEAD-1): an international, open-label, phase 2 trial	NCT03132922
	Ishihara 2023	A phase 1 trial of NY-ESO-1-specific TCR-engineered T-cell therapy combined with a lymph node-targeting nanoparticulate peptide vaccine for the treatment of advanced soft tissue sarcoma	NCT03462316
	Kohli 2021	IL-15 mediated expansion of rare durable memory T cells following adoptive cellular therapy	NCT04177021
	Pollack 2016	NY-ESO-1 Specific T Cells After Cyclophosphamide in Treating Patients with Advanced Synovial Sarcoma or Myxoid/​Round Cell Liposarcoma	NCT02059850

## Histology-agnostic Indications for Immunotherapy

Molecular features such as high microsatellite instability (MSI-H), deficient mismatch repair (dMMR), or high tumor mutational burden (TMB) can serve as predictive biomarkers for immunotherapy responsiveness, regardless of histology.

However, deficient mismatch repair and microsatellite instability are rare in STS [7]. Sarcomas are not considered part of the inherited Lynch syndrome tumor spectrum. However, pleomorphic subtypes such as UPS and pleomorphic rhabdomyosarcoma have been described to arise in individuals with Lynch syndrome, most often in carriers of MSH2 mutations [8]. Similarly, an elevated TMB (i.e. ≥10 mutations per megabase) is infrequently encountered, though has been described in angiosarcomas, often associated with ultraviolet mutational signatures [9] and pleomorphic dermal sarcomas. The latter are managed surgically with low rates of recurrence, thereby rarely requiring systemic therapy. UPS and ASPS may rarely exhibit high TMB.

As such, while high TMB and MSI-H/dMMR provide a histology-agnostic rationale for ICI use, their limited prevalence restricts their broad application in STS. 

## Histology-directed Indications for Immunotherapy

1. *Anthracycline-sensitive histologies*

Doxorubicin is a standard first-line agent for many STS subtypes, including liposarcoma, leiomyosarcoma, synovial sarcoma, angiosarcoma, and undifferentiated soft tissue sarcomas. It may be administered as monotherapy or in combination with ifosfamide (AIM), particularly in cases where rapid tumor shrinkage is a clinical priority [10]. These regimens offer significant improvements in patient outcome and provide a critical backbone upon which immunotherapeutic approaches are increasingly being evaluated in clinical trials and real-world practice.

A1.1  *Undifferentiated Pleomorphic Sarcoma*

UPS exemplifies one of several poorly differentiated sarcomas diagnosed after exclusion of other mesenchymal malignancy classifications. Clinical studies often group UPS, MFS, and other pleomorphic sarcomas with complex genomics together, as they share key genetic alterations (such as mutations in TP53, RB1, and CDKN2A), disease courses, and outcomes [11]. They most commonly arise in the extremities and trunk of older adults. Although most are sporadic, prior radiation and genetic syndromes such as Li-Fraumeni are recognized risk factors. Pazopanib, the tyrosine kinase inhibitor, is an FDA approved subsequent-line option for advanced or metastatic UPS and other non-adipocytic STS types, or as an alternative for patients who are not candidates for anthracyclines [12, 13].

Clinical trials have demonstrated significant promise for immunotherapy in UPS. The multicenter, single-arm phase 2 study SARC028 pioneered the use of pembrolizumab in UPS after identifying an objective response rate (ORR) of 40% in this subtype [14]. Additional analyses showed that higher densities of activated cytotoxic T cells and PD-L1–expressing tumor-associated macrophages were associated with response in UPS, suggesting an immunologically active tumor microenvironment [15].

Another key trial was Alliance A091401, a randomized, non-comparative phase 2 study of nivolumab alone or in combination with ipilimumab in 85 patients with advanced/metastatic sarcoma previously treated with at least one line of therapy. The combination of nivolumab plus ipilimumab demonstrated a higher ORR (16%; six patients, including those with UPS histology) versus nivolumab monotherapy (5%; two patients). Similar results were seen in an expansion cohort study of A091401 [16].

In the neoadjuvant setting, the randomized clinical trial SU2C-SARC032 compared the addition of pembrolizumab to standard preoperative radiotherapy and surgery versus radiotherapy and surgery alone in patients with stage III UPS or pleomorphic or de-differentiated liposarcoma (DDLPS) of the extremity. Pembrolizumab was administered as three neoadjuvant cycles and up to 14 adjuvant cycles. In a modified intention-to-treat analysis, the pembrolizumab group had significantly improved disease-free survival (DFS) (hazard ratio 0.61; 2-year DFS 67% vs. 52%) [17]. The trial established pembrolizumab plus radiotherapy and surgery as a promising option for high-risk, high-grade localized STS.

Dual ICI blockade with ipilimumab with nivolumab was also recently studied in NCT03307616, a randomized phase 2 trial that demonstrated that neoadjuvant nivolumab with or without ipilimumab and concurrent radiation therapy before surgery in patients with resectable UPS or DDLPS resulted in significant pathologic responses, particularly in UPS [18]. EA7222 is an ongoing phase 3 trial comparing chemoimmunotherapy to chemotherapy alone and is discussed in detail below.

A1.2  *Angiosarcoma*

Angiosarcoma may arise from cutaneous sites including the scalp and face, the breast (particularly post-radiation), deep soft tissues, as well as the liver and spleen. These tumors are rapidly-enlarging and present as bruise-like or nodular lesions that may bleed. Diagnosis is based on vasoformative architecture and immunoreactivity for endothelial markers (CD31, CD34). Other than single-agent doxorubicin, or more appropriately AIM in rapidly growing tumors, single-agent paclitaxel is a widely used front-line regimen in angiosarcoma especially for cutaneous angiosarcoma or angiosarcoma of the breast [6]. The gemcitabine and docetaxel combination is also considered for patients with high-volume or rapidly progressive disease.

As with UPS, ipilimumab plus nivolumab and pembrolizumab monotherapy have demonstrated objective responses in angiosarcoma. The combination of ipilimumab and nivolumab was investigated in the SWOG S1609 (DART) phase II trial, demonstrating an ORR of 25% in metastatic or unresectable angiosarcoma, with particularly high response rates (60%) in patients with cutaneous scalp or face primaries [19]. For pembrolizumab, a retrospective cohort study of 25 patients with angiosarcoma reported an ORR of 18% and a disease control rate of 59%, with a median progression-free survival (PFS) of 6.2 months [20]. Nivolumab monotherapy data is less convincing, evaluated in a phase II trial (AngioCheck) in pretreated cutaneous angiosarcoma, showing a centrally reviewed ORR of 13% and a median PFS of 59 days [21].

The multicenter phase II clinical trial Alliance A091902 evaluated the combination of cabozantinib (40 mg orally daily) and nivolumab (480 mg IV every 4 weeks) in patients with advanced angiosarcoma who had previously received taxane-based therapy. The ORR was 62% (13/21 patients; 95% CI: 38–82%), with responses observed in both cutaneous (58%) and non-cutaneous (67%) disease [22]. Median PFS was 9.6 months, and median overall survival (OS) was 20.5 months. These results indicate that cabozantinib plus nivolumab demonstrates significant antitumor activity and an acceptable safety profile in taxane-pretreated advanced angiosarcoma.

A1.3  *Synovial Sarcoma*

Synovial sarcoma can arise from any serosal surface, including the pleura and renal capsule, but most often arises near large joints of the extremities in adolescents and young adults. The defining genomic alteration is the SS18:SSX fusion, t(X;18) [23]. As synovial sarcoma is generally sensitive to ifosfamide, AIM is typically used, but other systemic therapy options include pazopanib, trabectedin, gemcitabine and docetaxel, and ifosfamide monotherapy [6]. In contrast to UPS and angiosarcoma, where ICI have demonstrated clinically meaningful activity, these agents have demonstrated limited effectiveness on synovial sarcoma [24, 25].

However, the high expression of cancer testis antigens in synovial sarcoma has been leveraged for treatment. These are highly immunogenic self-proteins involved in fetal development but not normally expressed outside of the testis or placenta in healthy adults [26]. Afamitresgene autoleucel (afami-cel) is a genetically-engineered T-cell therapy that targets the cancer testis antigen MAGE-A4, recently approved for refractory or relapsed disease. Another, letetresgene autoleucel (lete-cel) targets the cancer testis antigen NY-ESO-1, and has demonstrated clear clinical activity [27].

Unlike CAR-T, these engineered T cells receptors (TCR) target intracellular tumor-associated antigens that are processed and presented on tumor cells by HLA-A*02:01 or related alleles, which is why they are HLA subtype restricted. Patients typically undergo lymphodepleting chemotherapy with cyclophosphamide and fludarabine, followed by TCR infusion. Response rates in this population are approximately 40–60%, with durable responses in a subset [28, 29]. Predictive biomarkers include higher pre-infusion IL-15, greater numbers of infused effector memory CD8 + T cells, and robust in vivo T-cell expansion. Other therapies targeting these antigens, such as cancer vaccines, have been examined in a phase II randomized study with some evidence of clinical benefit [25].

A1.4  *Liposarcoma*

Liposarcomas are adipocytic STS subtypes. DDLPS is the most common, driven by MDM2/CDK4 amplification. Myxoid/round cell liposarcoma (MRCLS) is defined by the FUS-DDIT3 fusion. Pleomorphic liposarcoma (PLS) represents another ultra-rare liposarcoma.

For DDLPS, the NCCN recommends first-line systemic therapy with doxorubicin, with or without ifosfamide, but response rates are lower than in MRCLS. Trabectedin and eribulin are supported as a category 1 recommendations for liposarcoma by the NCCN, with trabectedin particularly important for MRCLS and eribulin for PLS [6].

Although data from the phase II SARC028 trial suggested that pembrolizumab was active in DDLPS, there remains controversy regarding the role it should play in management [14]. Nonetheless, NCCN guidance indicates that PD-1 inhibitors with or without CTLA-4 inhibition is reasonable for recurrent or metastatic DDLPS. The immune microenvironment does appear to play a key role in DDLPS prognosis. Elevated CD4⁺ T-cell levels are associated with improved outcomes, whereas increased CD14⁺ cells and M2 macrophages correlate with adverse outcomes [30]. Wagner et al. (2022) reported data from an early phase trial combining avelumab and trabectedin for advanced liposarcoma and leiomyosarcoma. While the study did not achieve its primary endpoint of ORR, PFS compared well with previous trabectedin trials, supporting a need for additional research in this domain [31]. NCT05694871 and NCT04438824 (see Table 1.) are ongoing trials exploring palbociclib in combination with immunotherapy in cases of DDLPS.

MRCLS is generally more chemo-sensitive than DDLPS. Long-term disease control using trabectedin is achievable [32]. It is also notably radiosensitive, and preoperative radiotherapy is frequently used to facilitate resection and improve local control [33]. T-cell therapy is under investigation for MRCLS due to consistently high cancer testis antigen expression [34]. D’Angelo, at al. reported an ORR ranging from 20 to 40% of lete-cel in MRCLS, with varying rates based on dosing [35]. Though the response rate was lower than in synovial sarcoma, afami-cel was also studied in the phase 2 SPEARHEAD-1 trial (NCT04044768). An ORR of 25% (two of eight; 95% CI 3–65) versus 39% (19 of 52; 95% CI 24–51) in synovial sarcoma was observed [28]. Finally, IMA203, an autologous TCR T-cell therapy directed against PRAME is being evaluated in a phase 1 trial (NCT03686124) for HLA-A*02-positive patients with PRAME-positive advanced solid tumors, including sarcomas. Interim data indicate promising anti-tumor activity and safety [36].

AI.5  *Leiomyosarcoma*

Leiomyosarcoma (LMS) can arise in a variety of different primary sites, including the uterus in approximately one-third of cases, but also retroperitoneal or deep soft tissue. These tumors are genomically complex, with frequent TP53, RB1, and ATRX mutations [37]. Diagnosis is based on spindle cell morphology with smooth muscle markers (desmin, SMA). First-line systemic therapy for non-uterine LMS is doxorubicin, either as monotherapy or in combination with trabectedin. Alternative effective regimens include doxorubicin plus dacarbazine or AIM [38]. Gemcitabine plus docetaxel can also be used in the front line, particularly in patients unable to tolerate anthracyclines. Other therapies commonly used for refractory disease include pazopanib.

Immunotherapy in non-uterine LMS has shown limited efficacy. Prospective trials such as SARC028 (pembrolizumab) and Alliance A091401 (nivolumab with or without ipilimumab) demonstrated markedly low response rates in LMS (generally < 10%) [14, 16]. Combination strategies (e.g., lenvatinib plus pembrolizumab, NCT04784247) are under investigation and further discussed below. The phase 2 trial of trabectedin plus nivolumab in pretreated advanced LMS by the German Interdisciplinary Sarcoma Group (GISG-15, NiTraSarc) demonstrated indeterminate efficacy data [39]. However, more recent results from the Spanish Sarcoma Group demonstrate that the combination of doxorubicin, dacarbazine, and nivolumab as first-line therapy for advanced LMS is feasible, well tolerated, and with encouraging clinical activity (ORR 56.5% partial response, with a 6-month PFS rate of 80%) [40].

A1.6  *Epithelioid sarcoma*

Epithelioid sarcoma (ES) typically affects the distal extremities of young adults (classic type) or proximal limbs/groin (proximal type). Clinically, it presents as a firm, slow-growing, often ulcerating nodule, and is characterized by loss of SMARCB1/INI1 expression. Diagnosis relies on epithelioid and spindle cell morphology, keratin/EMA positivity, and INI1 loss. Similarly, systemic therapy options for ES include first-line anthracycline-based regimens and gemcitabine-based regimens. The EZH2 inhibitor tazemetostat is approved for advanced ES [41]. For ES, prospective immunotherapy data are extremely limited, and no significant benefit has been demonstrated in published trials to date.

B. *Anthracycline-ambivalent or resistant histologies*

B1.1  *Alveolar soft part sarcoma*

Alveolar soft part sarcoma (ASPS) most commonly arises in the deep soft tissues of the extremities in adolescents and young adults. Metastasis is common at diagnosis. The pathophysiology is defined by the ASPSCR1-TFE3 fusion from t(X;17) (p11; q25), driving MET overexpression and angiogenesis [42]. Cytotoxic chemotherapy is generally ineffective in ASPS. Atezolizumab was evaluated in a multicenter, phase 2 trial (NCT03141684) and is now standard for advanced ASPS, based on robust and durable responses. It was associated with a 37% ORR, and a median duration of response of 24.7 months, and median PFS of 20.8 months [43]. Responses were observed in both intracranial and extracranial disease.

The combination of pembrolizumab with axitinib. the VEGF tyrosine kinase inhibitor (TKI), shows promising efficacy. Pembrolizumab monotherapy was initially studied in the AcSé-pembrolizumab basket trial (NCT03012620) [44] demonstrating an ORR at 12 weeks was 6.2% across all rare sarcoma subtypes. The ICI-TKI combination was evaluated in a single-center, phase 2 trial (NCT02636725) enrolling 12 patients with ASPS. It was generally well-tolerated, and the 3-month PFS rate in ASPS was 72.7% [45]. Responses were also identified in patients with heavy tumor burden or rapid progression.

B1.2  *Clear cell sarcoma*

Clear cell sarcoma (CCS) typically presents in the deep soft tissues of the distal extremities of young adult as a slow-growing mass. CCS pathophysiology involves the EWSR1-ATF1 fusion from t(12;22), leading to melanocytic differentiation [46]. The NCCN recommends doxorubicin-based regimens as first-line for advanced disease, but responses are poor [47]. Sunitinib and MET kinase inhibitors are under investigation. Nivolumab and pembrolizumab have shown limited activity in prospective trials, with response rates < 10% [48]. MART-1 may represent an actionable immunogenic target due to reported high expression in CCS [46] as it has proven in melanoma.

B1.3  *Malignant peripheral nerve sheath tumors*

Malignant peripheral nerve sheath tumors (MPNST) are typically rapidly-enlarging, often painful masses that occur in the extremities, trunk, or along major nerve plexuses, especially in patients with neurofibromatosis type 1 (NF1) [49]. Diagnosis is based on spindle cell morphology, focal S100 positivity, and loss of H3K27me3. Although often resistant, anthracycline-based chemotherapy remains the most commonly used first-line systemic therapy for advanced or metastatic disease. Other chemotherapeutics to consider for MPNST include gemcitabine, docetaxel, and dacarbazine. The Alliance A091401 trials included MPNST patients but minimal activity was observed for single-agent PD-1 blockade. However, a recent prospective, histology-specific trial (NCT04784247) of lenvatinib plus pembrolizumab showed a signal of potential benefit potentially warranting further investigation [50].

B1.4  *Other rare STS*

Other ultra-rare STS subtypes lack significant published data on efficacy of immunotherapy. Perivascular epithelioid cell tumor (PEComa) most often arises in the retroperitoneum, pelvis, uterus, or soft tissues. Pathophysiology is driven by TSC1/TSC2 mutations leading to mTOR pathway activation [51]. The NCCN recommends mTOR pathway inhibition via nab-sirolimus as first-line for advanced disease. Cytotoxic chemotherapy and VEGF-TKIs are less effective and reserved for later lines. Immunotherapy is investigational, with no established benefit [52].

Epithelioid hemangioendothelioma (EHE) of soft tissue is another rare tumor of vascular origin, most often affecting middle-aged adults, with no clear predisposing risk factors. It is defined by pathognomonic gene fusions (WWTR1-CAMTA1 or YAP1-TFE3) that drive tumorigenesis and result in variable clinical behavior ranging from indolent to aggressive disease [53]. Aggressive disease may be difficult to treat with standard therapies, and any role for immunotherapy remains unclear.

## Notable On-going Trials

Current research in STS immunotherapy focuses on refining patient selection through the identification of effective biomarkers, as well as exploring rational combinations with chemotherapy, TKIs, other targeted therapeutics and emerging immune modulators.

Chemoimmunotherapy combinations remain a key area of interest. The phase III EA7222 trial randomizing to doxorubicin alone or in combination with pembrolizumab (NCT06422806) is investigating whether concurrent immune checkpoint blockade enhances the antitumor effect of anthracyclines in metastatic UPS, related sarcomas, and DDLPS [54]. The SAINT study (NCT03138161) [55] is investigating first-line trabectedin with dual checkpoint blockade (ipilimumab and nivolumab), highlighting potential in triplet regimens.

There may be synergy between immunotherapy and other targeted therapeutics. The PEMBROCABOSARC study (NCT05182164) is investigating the combination of pembrolizumab with cabozantinib, a multitargeted TKI with antiangiogenic and immunomodulatory properties, across multiple histologies [56]. Similarly, studies pairing palbociclib (a CDK4/6 inhibitor) with PD-1 immune checkpoint inhibitors cemiplimab (NCT05694871) [57] or retifanlimab (NCT04438824) are also underway [58]. Pre-clinical studies have described multiple mechanisms for synergy. CDK4/6 inhibitors can enhance tumor immunogenicity by upregulating MHC class I expression and antigen-presentation machinery while preferentially suppressing FOXP3⁺ regulatory T cells and sparing CD8⁺ effector T cells [59]. In addition, CDK4/6 inhibition promotes memory-like T-cell differentiation, supporting more durable antitumor immune responses [60].

Novel immune-modulatory agents are also emerging. These include next-generation immune checkpoint inhibitors. The early phase trial NCT03860272 evaluated the combination of botensilimab (an Fc-enhanced anti-CTLA-4 antibody) and balstilimab (anti-PD-1) in patients with advanced, heavily pre-treated sarcomas and identified promising efficacy and safety [61]. POD1UM-101 was a first-in-human study of PD-1 inhibitor retifanlimab [62], and NCT03475251 was a phase 1 study of PD-1 inhibitor nofazinlimab [63].

The TRUST study (NCT04874311) [64] combines doxorubicin with bintrafusp alfa, a bifunctional agent that works through colocalized targeting of PD-L1 and TGF-β. Once bound to tumor cells via PD-L1, bintrafusp alfa induces internalization and lysosomal degradation of TGF-β, actively depleting it from the tumor microenvironment rather than simply blocking it. This has been shown to have multiple effects including increased tumor-infiltrating lymphocytes, modulation of macrophage phenotype from M2 to M1, reduced regulatory T cells and myeloid-derived suppressor cells [65].

NCT04242238 was a phase I study that corroborated safety of the combination of vimseltinib and avelumab (a PD-L1 inhibitor) in patients with unresectable or metastatic sarcoma [66]. Immunosuppressive M2 macrophages are polarized, in part by CSF1, leading to suppression of CD8 + T cells, recruitment of regulatory T cells, and resistance to immune checkpoint blockade. Vimseltinib (DCC-3014) is an orally active small-molecule inhibitor with > 100-fold selectivity for CSF1R. The sensitization of STS to ICI is likely to be further explored through additional research in myeloid compartment modulation [67].

Cellular and antigen-targeted immunotherapies are also expanding. ADP-A2M4CD8 is a next-generation TCR targeting MAGE-A4 that additionally incorporates a CD8α co-receptor, which enhances T-cell receptor signaling in CD4⁺ T cells, thereby augmenting their activation, cytotoxic potential, and functional persistence [68]. Investigations into tumor-infiltrating lymphocyte (TIL) therapy [69], cytokine support (e.g., IL-15–driven expansion), and vaccine-based approaches may identify further potential for personalized, durable immune activation [70], complementing chemotherapy and immune checkpoint inhibitor combinations and reinforcing the shift toward precision immunotherapy in STS.

Tertiary lymphoid structures (TLS) are ectopic lymphoid aggregates that arise in non-lymphoid tissues under conditions of chronic inflammation, including within tumors. The presence of TLS has been shown to predict improved responses to ICI [15], as demonstrated across multiple clinical studies, including the landmark PEMBROSARC trial. Consequently, several preclinical and early-phase clinical investigations are evaluating therapeutic strategies to induce TLS formation in TLS-negative tumors. These approaches include the development of novel biomaterial-based interventions to deliver cytokines and chemokines [71, 72].

METROMAJX was a phase II trial evaluating intratumoral injection of oncolytic virus JX-594 combined with metronomic cyclophosphamide and avelumab in patients with immunologically “cold” advanced soft tissue sarcomas lacking tertiary lymphoid structures [73]. Although the regimen was well tolerated, with predominantly grade 1 fatigue and fever, only 1 of 14 evaluable patients was progression-free at 6 months, and the study did not meet the first-stage Simon design efficacy threshold. Correlative analyses demonstrated increased intratumoral CD8⁺ T-cell density and upregulation of immune biomarkers, including CXCL10, indicating biologic modulation of the tumor microenvironment despite limited clinical efficacy.

Other oncolytic viruses are also being studied. NCT03886311 was a phase II trial that investigated intratumoral talimogene laherparepvec combined with trabectedin and nivolumab in previously treated advanced sarcomas [74]. Among 39 evaluable patients, the 12-month progression-free survival rate was 36.7%, with a disease control rate of 84.6% and median overall survival of 19.3 months.

## Conclusion

The management of STS remains challenged by significant heterogeneity across subtypes, necessitating tailored therapeutic strategies. While ICI have demonstrated efficacy in select sarcoma subtypes, ongoing efforts to expand their utility through rational combination strategies—such as with chemotherapy, targeted agents, and immune modulators—are promising. Advances in our understanding of tumor resistance mechanisms, immune exhaustion, and the tumor microenvironment will be critical to broadening the impact of immunotherapy. Engineered T-cell therapies are also emerging as a compelling frontier in sarcoma, outpacing other solid tumors. Moving forward, the development of reliable predictive biomarkers will be essential to guide patient selection and optimize outcomes. Finally, the integration of immune-sequencing, spatial transcriptomics, and gene-expression profiling into early-phase trial design is likely to define future immunotherapy paradigms.

## Key References

- Mowery YM et al., 2024. Pembrolizumab, radiation therapy, and surgery versus radiation therapy and surgery for stage III soft tissue sarcoma (SU2C-SARC032). Lancet.
  - ○ This randomized clinical trial demonstrated a disease-free survival benefit with the addition of pembrolizumab to standard neoadjuvant radiation and surgery in high-risk localized UPS and DDLPS, providing strong evidence for integrating immunotherapy into curative-intent treatment paradigms.
- Roland CL et al., 2024. Neoadjuvant immune checkpoint blockade in undifferentiated pleomorphic sarcoma and dedifferentiated liposarcoma. Nature Cancer.
  - ○ This study showed that neoadjuvant immune checkpoint blockade, particularly in UPS, can induce meaningful pathologic responses and immune remodeling, supporting further exploration of immunotherapy in earlier disease settings.
- Wilky BA et al., 2025. Botensilimab plus balstilimab in relapsed/refractory metastatic sarcomas. Journal of Clinical Oncology.
  - ○ This study provides early but compelling evidence that next-generation, Fc-enhanced CTLA-4 blockade combined with PD-1 inhibition can be efficacious in heavily pretreated sarcoma patients, supporting further development of optimized checkpoint antibodies beyond first-generation agents.
- D’Angelo SP et al., 2024. Afamitresgene autoleucel for advanced synovial sarcoma and myxoid round cell liposarcoma (SPEARHEAD-1). Lancet.
  - ○ This international phase II trial established engineered T-cell receptor therapy targeting MAGE-A4 as a highly active treatment in synovial sarcoma, marking a major advance for cellular immunotherapy in solid tumors.
- Toulmonde M et al., 2024. Reshaping the tumor microenvironment of cold soft-tissue sarcomas with oncolytic viral therapy. Molecular Cancer.
  - ○ This phase II study demonstrated biologic immune modulation in immunologically “cold” sarcomas using oncolytic viral therapy in combination with chemoimmunotherapy, highlighting innovative approaches to convert resistant tumors into immunotherapy-responsive disease.

## Data Availability

No datasets were generated or analysed during the current study.
